# A Validated Preharvest Sampling Simulation Shows that Sampling Plans with a Larger Number of Randomly Located Samples Perform Better than Typical Sampling Plans in Detecting Representative Point-Source and Widespread Hazards in Leafy Green Fields

**DOI:** 10.1128/aem.01015-22

**Published:** 2022-11-15

**Authors:** Jorge Quintanilla Portillo, Xianbin Cheng, Alexandra M. Belias, Daniel L. Weller, Martin Wiedmann, Matthew J. Stasiewicz

**Affiliations:** a Department of Food Science and Human Nutrition, University of Illinois at Urbana-Champaign, Champaign, Illinois, USA; b Department of Food Science, Cornell University, Ithaca, New York, USA; c Department of Biostatistics and Computational Biology, University of Rochester, Rochester, New York, USA; The Pennsylvania State University

**Keywords:** food safety sampling, preharvest produce safety, simulation, leafy greens

## Abstract

Commercial leafy greens customers often require a negative preharvest pathogen test, typically by compositing 60 produce sample grabs of 150 to 375 g total mass from lots of various acreages. This study developed a preharvest sampling Monte Carlo simulation, validated it against literature and experimental trials, and used it to suggest improvements to sampling plans. The simulation was validated by outputting six simulated ranges of positive samples that contained the experimental number of positive samples (range, 2 to 139 positives) recovered from six field trials with point source, systematic, and sporadic contamination. We then evaluated the relative performance between simple random, stratified random, or systematic sampling in a 1-acre field to detect point sources of contamination present at 0.3% to 1.7% prevalence. Randomized sampling was optimal because of lower variability in probability of acceptance. Optimized sampling was applied to detect an industry-relevant point source [3 log(CFU/g) over 0.3% of the field] and widespread contamination [−1 to −4 log(CFU/g) over the whole field] by taking 60 to 1,200 sample grabs of 3 g. More samples increased the power of detecting point source contamination, as the median probability of acceptance decreased from 85% with 60 samples to 5% with 1,200 samples. Sampling plans with larger total composite sample mass increased power to detect low-level, widespread contamination, as the median probability of acceptance with −3 log(CFU/g) contamination decreased from 85% with a 150-g total mass to 30% with a 1,200-g total mass. Therefore, preharvest sampling power increases by taking more, smaller samples with randomization, up to the constraints of total grabs and mass feasible or required for a food safety objective.

**IMPORTANCE** This study addresses a need for improved preharvest sampling plans for pathogen detection in leafy green fields by developing and validating a preharvest sampling simulation model, avoiding the expensive task of physical sampling in many fields. Validated preharvest sampling simulations were used to develop guidance for preharvest sampling protocols. Sampling simulations predicted that sampling plans with randomization are less variable in their power to detect low-prevalence point source contamination in a 1-acre field. Collecting larger total sample masses improved the power of sampling plans in detecting widespread contamination in 1-acre fields. Hence, the power of typical sampling plans that collect 150 to 375 g per composite sample can be improved by taking more, randomized smaller samples for larger total sample mass. The improved sampling plans are subject to feasibility constraints or to meet a particular food safety objective.

## INTRODUCTION

The production of leafy greens in open fields makes them susceptible to contamination from bacteria in animal feces, water, and soil (1–6), among other sources. Once harvested, leafy greens usually undergo minimal processing and are often consumed raw. The lack of a kill step during postharvest processing makes preharvest bacterial contamination a food safety risk that should be managed.

To address this risk, one tool is preharvest sampling and testing to identify pathogens in contaminated produce in production fields, used in combination with good agricultural practices. Publicly available preharvest sampling recommendations and requirements include (i) microbiological criteria and sampling plans published by the International Commission on Microbiological Specifications for Foods (ICMSF) (7, 8) and (ii) requirements for market access, such as import requirements established by the Canadian Food Inspection Agency, that require growers to certify that their product was sampled, tested, and does not contain detectable levels of Escherichia coli O157:H7 (9).

The development of statistically powerful plans to detect preharvest pathogens is difficult, since contamination typically occurs at a low prevalence or level (10). For example, when 1% of the collected units are contaminated, testing 60 sample units would detect contaminated product 45% of the time (8, 11). Reliable detection of contaminated produce at low prevalence (<1%) during routine preharvest produce testing would require collection of many more grab samples for composite testing.

Further, different mechanisms of contamination may produce different patterns of contamination. For example, wildlife feces may contaminate produce only within a small distance from the feces (e.g., 2 m) (4, 12), whereas irrigation with contaminated water may more uniformly inoculate a field (1, 6, 13, 14). In addition, produce may be contaminated by pathogens endemic to the field environment, resulting in sporadic, low-level contamination (15–17). The most efficient sampling plan depends on which type of contamination has occurred (18). For example, systematic sampling (e.g., Z-pattern sampling, walking a field in a Z pattern up one edge, down to the opposite edge, and up that edge) is done because it is easier to collect samples than by sampling at random locations, but it may be more likely to miss point sources of contamination by leaving a part of the field unsampled (19). As such, sampling plans should be developed that account for contamination events that are relatively likely for the given operation.

The objective of this study was to address a need for improved sampling plans for preharvest field testing. This paper will meet this need by developing and validating a produce field simulation, which can be used to evaluate different approaches to preharvest sampling in produce fields (e.g., different sampling patterns, more produce sample grabs, larger total sample mass). These simulations will provide data for users to develop preharvest sampling plans optimized to detect specific types and levels of field contamination that can be applicable irrespective of the pathogen.

## RESULTS

### The sampling simulation was validated against published theoretical detection probabilities.

**FIG 1:**
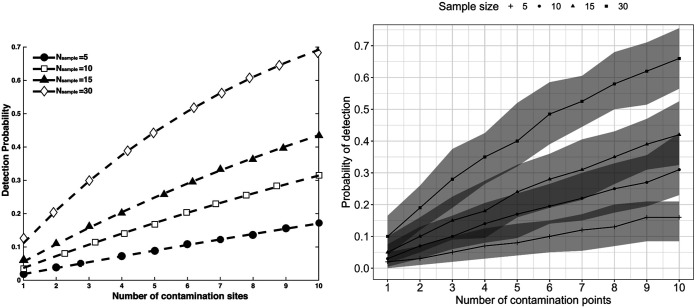
Simulation model validation against published theoretical detection probabilities from a leafy greens sampling study. (Left) Published detection probabilities for simulation of sampling in an 18- by 15-m field contaminated at 1 to 10 sites (equivalent to a prevalence level between 0.3% and 3.7%) and sampled by 5 to 30 simple random samples. The figure was reproduced with permission from the publisher of Xu and Buchanan (19). (Right) Validation of the sampling scenarios of the left panel by our simulation model with 10,000 iterations at each sampling scenario. Each point in the curve represents the median probability of detection, and the shaded area represents the range between the 2.5th and 97.5th percentiles.

To validate the simulation, we compared the probability of detection to the theoretical detection probability for sampling a 15- by 18-m field reported in a previous sampling study (Fig. 1). By visual inspection, both the published theoretical detection probability and our simulated probability of detection showed a trend of increasing detection probability from ~1% to ~70% with an increasing number of contamination points and increasing sample sizes. A closer examination revealed that all the theoretical detection probabilities fell within the corresponding 2.5th to 97.5th percentile-simulated range of probability of detection. Therefore, our simulation can accurately predict the detection probabilities from a previous study. In conclusion, our simulation model was successfully validated against published theoretical detection probabilities.

### The sampling simulation was validated against experimental field data specifically collected for model validation.

For the point source contamination trials, rifampin-resistant generic E. coli (rE. coli) was recovered from 17 and 7 of 228 samples from the first and second trial, respectively. In the first trial (PS1), PCR testing of presumptive positive isolates confirmed 3/17 matched one of the inoculum strains. Therefore, naturally occurring rE. coli was isolated from the other 14/17 samples. Naturally occurring rE. coli was also previously found in soil, irrigation water, and produce in previous studies at this location (6, 20). For the second trial (PS2), all 7/7 presumptive positive isolates were confirmed as a positive match to one of the inoculum strains.

For the systematic contamination trials, r*E. coli* was recovered from 139/228 samples in both trials (SS1 and SS2; from different samples in each trial). PCR confirmation testing of 13/13 randomly selected isolates in both trials showed that all isolates matched the inoculum strains. Given that no samples failed to match the inoculum strains, there was 95% confidence that <20% of the samples were positive due to naturally occurring rE. coli. Therefore, at least 80% of the total positive samples recovered contained inoculum strains.

For the sporadic contamination trials, r*E. coli* was recovered from 10/228 samples in SP1, which was resampled 6 days postinoculation, and 18/228 samples in SP2, which was resampled 5 days postinoculation. In SP1, 2/10 isolates were confirmed inoculum strains. Because only 2/10 isolates from SP1 were the inoculum strains, this suggested that 8/10 other rE. coli strains represented a background population with increased environmental survival relative to the inoculum strain; still, the fact that presumptive positives decreased from 139 to 10 in this trial showed die-off of at least the inoculum strain (and background strain, which was <20% of the population in the systematic contamination and likely survived better and became relatively more abundant in the sporadic contamination sampling). In SP2, all 18/18 isolates were confirmed inoculum strains. These experimental data confirmed there was drastic r*E. coli* die-off in the 4 to 5 days between the systematic contamination sampling (SS1 and SS2), where 139 samples were presumptive positive, and sporadic contamination sampling (SP1 and SP2), where 10 and 18 samples were presumptive positive.

**FIG 2:**
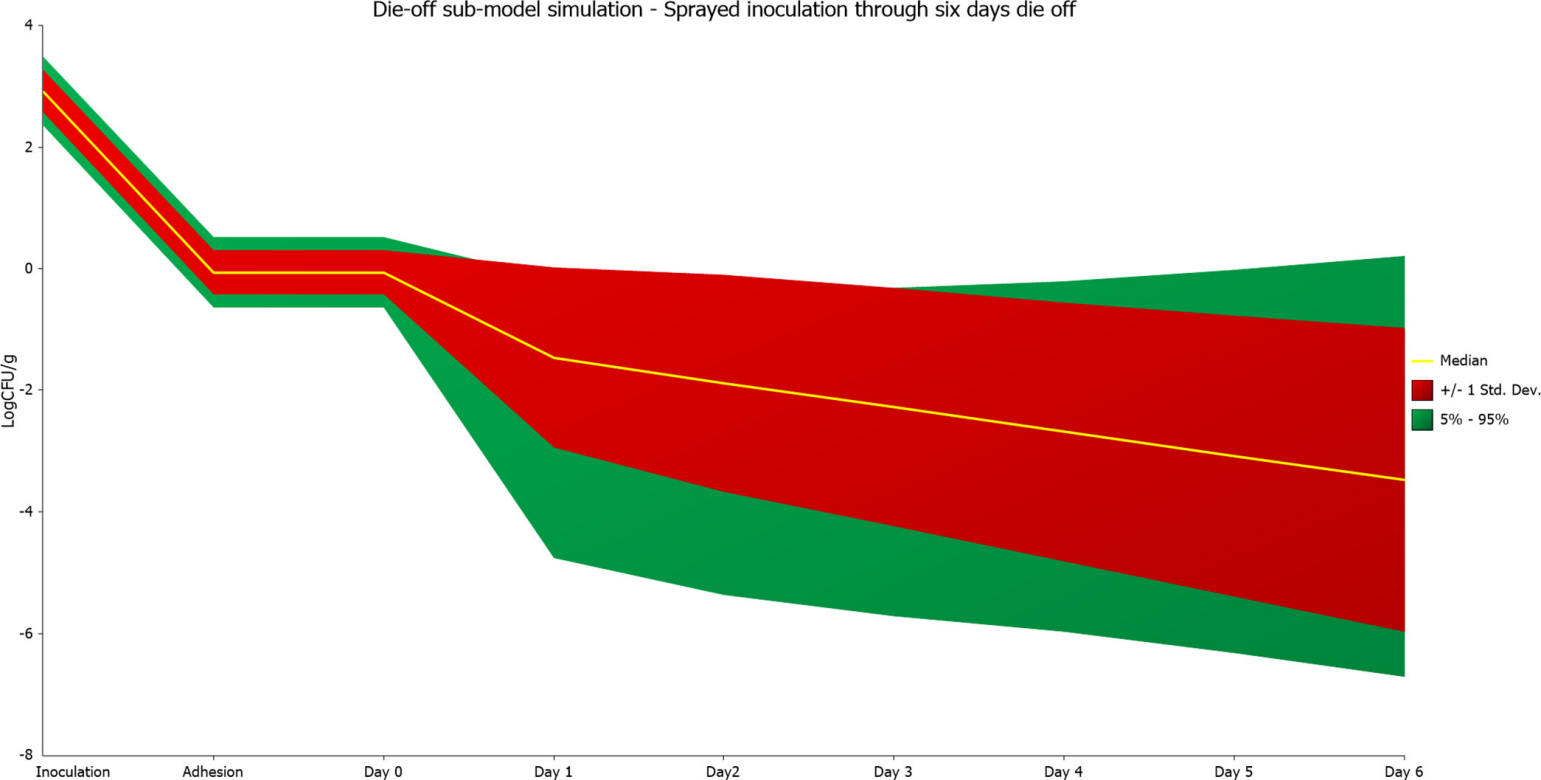
Submodel of change in bacterial concentration from inoculation, adhesion, and die-off over 6 days. Sprayed inoculation levels and variability were experimentally measured during systematic contamination trials and used as input. Adhesion reduction was adjusted to result in a concentration of −1.47 log(CFU/g) needed in field to recover 139 positive samples, consistent with the systematic contamination results (Fig. 3). Biphasic die-off parameters were obtained from Belias et al., 2020 (20). Simulation was performed using @Risk 8.0 with input parameters listed in Table 3. Predicted concentrations at time of sampling are listed on Table 1. Results are from 10,000 iterations.

Prior to validation against experimental data, it was necessary to predict the hazard concentration at time of sampling and then simulate the stratified random sampling events. The model of spraying, adhesion, and die-off showed an approximately 3-log reduction in inoculum concentration after spraying (adhesion parameter), followed by a die-off, and then slower change (biphasic die-off model) (Fig. 2). The output of this die-off model and other key field trial simulation parameters listed in Table 1 were used as inputs in each field trial simulation. This die off model was based on experimental data for die-off of rifampin-resistant E. coli in spinach in New York (20). Previous studies suggested that other foodborne pathogens, e.g., Salmonella and E. coli O157:H7, might have different capacities to adapt and survive in produce environments (21–25). Hence, the die-off model may not be applicable to other organisms and/or produce commodities.

**TABLE 1:** Simulation parameter inputs and outputs for inoculated field trials validation

Field trial	Field dimension (m)	Contamination geometry	No. of contamination points	Sampling plan^*a*^	Individual sample mass (g)	Contamination level distribution^*b*^ [log(CFU/g)]	Median predicted inoculum concn^*c*^ [log(CFU/g)]	2.5–97.5 percentile inoculum concen [log(CFU/g)]^*c*^
Point source contamination 1	28.5 × 5.3	Point source (spread radius, 1.9 m)	1	STRS	25	Gumbel (L = 0.9987, S = 0.931)	0.75	−3.37 to 1.99
Point source contamination 2	28.5 × 5.3	Point source (spread radius, 1.9 m)	1	STRS	25	Gumbel (L = 1.0215, S = 0.9328)	0.78	−2.27 to 1.91
Systematic contamination 1	28.5 × 5.3	Area	Not applicable	STRS	25	Gumbel (L =−1.19, S = 1.0107)	−1.48	−5.83 to 0.01
Systematic contamination 2	28.5 × 5.3	Area	Not applicable	STRS	25	Gumbel (L =−0.994, S = 0.9327)	−1.24	−5.37 to −0.005
Sporadic contamination 1	28.5 × 5.3	Area	Not applicable	STRS	25	Logistic (L =−3.367, S = 1.152)	−3.43	−7.22 to 1.23
Sporadic contamination 2	28.5 × 5.3	Area	Not applicable	STRS	25	Logistic (L =−2.804, S = 1.0216)	−2.82	−6.61 to 0.86

a STRS, stratified random sampling. Each field trial stratification consisted of 228 strata. Each stratum was sampled individually, for a total of 228 total samples.

b Best fit distribution of the simulated data from die-off mode, determined by lowest Akaike information criterion value. L, location; S, scale.

c As sampled from the distribution fit for each field trial.

**FIG 3:**
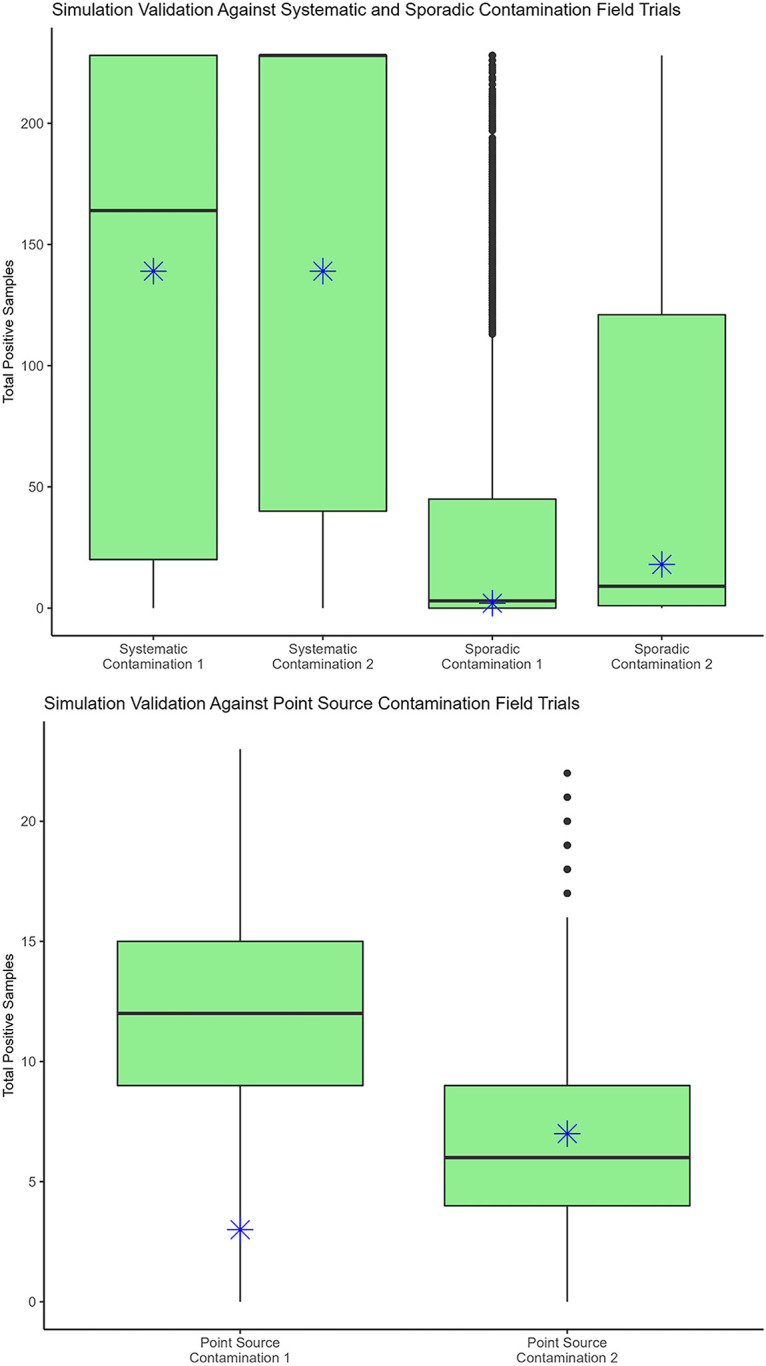
Simulation validation against inoculated field trials. (Top left) Simulated sampling detection of the inoculum in a systematically contaminated field at a relatively high concentration [~3 log(CFU/mL)] following 1 day of inoculation and die-off. Simulated positive samples had 2.5th to 97.5th percentiles that ranged from 17 to 228 across both trials. (Top right) Simulated sampling detection of the inoculum in a low-level, sporadic contamination event across the entire field at a concentration due to die-off following 5 to 6 days of inoculation. Simulated positive samples had 2.5th to 97.5th percentiles ranging from 0 to 228 across both trials. (Bottom) Simulated sampling detection of the inoculum in a field with a single point source of contamination at a relatively high concentration at the center [~5 log(CFU/mL)] of an area of 1.9-m radius (12), following 1 day of inoculation. Simulated positive samples had 2.5th to 97.5th percentiles that ranged from 1 to 19 across both trials. Total positive samples recovered from field trials are represented with an asterisk inside each simulated trial boxplot. Simulated medians of total numbers of positive samples are presented as horizontal lines, the boxplot shows the IQR, upper whiskers are the calculated Q3 max value +1.5× IQR, lower whiskers are the calculated Q1 min value −1.5× IQR, and outliers are the points. Each sampling scenario was iterated for 100 contamination patterns and 100 sampling patterns (*n* = 10,000 iterations).

For systematic contamination, simulated positive samples had 2.5th to 97.5th percentile ranges from 17 to 228, with a simulated median at 164; the experimentally observed 139 positives was close to the median simulation for SS1, as expected due to the calculated adjustment for log reduction due to the adhesion parameter calibrated for that trial data. This now-well-calibrated model was then used to simulate the other trials. The median of simulated positive samples from SS2 was higher (median, 228), likely due to the slightly higher and less-variable inoculum sprayed but keeping the same adhesion and die-off model (Fig. 3, top left).

For sporadic contamination, simulated positive samples had 2.5th to 97.5th percentile ranges from 0 to 228, with an interquartile range (IQR) from 0 to 118 for positive samples across both trials and simulated medians of 2 and 9 for SP1 and SP2, respectively. The experimentally observed 2 and 18 positive samples were within the IQR and close to the median simulation (Fig. 3, top right). For point source contamination, simulated positive samples had 2.5th to 97.5th percentiles ranging from 1 to 19, with an IQR of 4 to 15 positive samples across both trials and simulated medians of 12 and 6 for PS1 and PS2, respectively. The experimentally observed 3 and 7 samples fell within the 2.5th to 97.5th percentile simulation range and in one trial were very close to the simulated median (Fig. 3, bottom). In summary, all experimental data fell within 95th percentile range of our simulations; therefore, the model was considered experimentally validated for this high-resolution stratified random sampling process.

### Simulation predicted that simple and stratified random sampling had less variability than systematic sampling in power to detect point source contamination.

**FIG 4:**
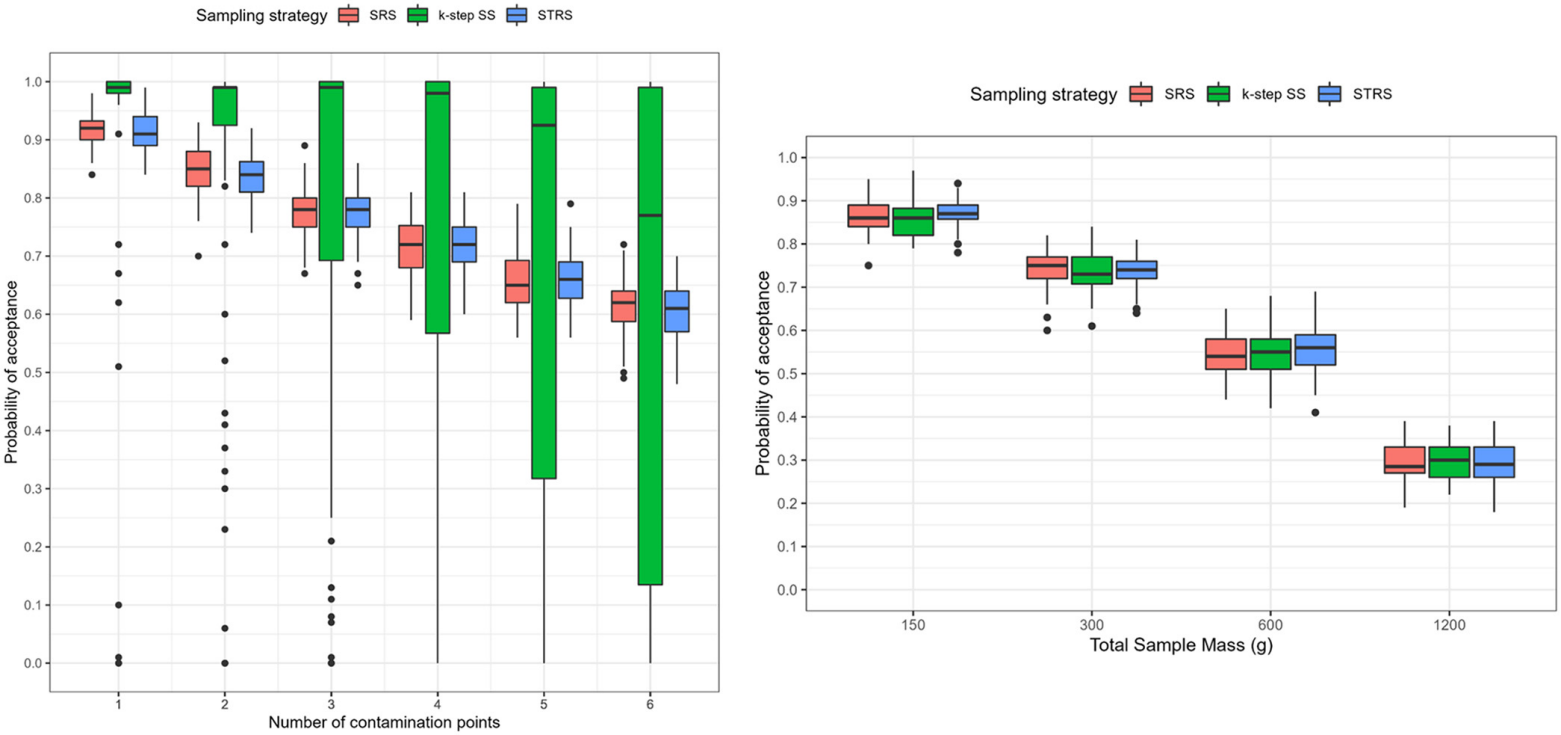
Analysis of the performance of sampling patterns to detect representative point source and widespread hazards in 1-acre generic fields. (Left) Probability of acceptance for three sampling strategies (SRS, STRS, and k-step SS) applied to detect contamination from 1 to 6 point sources of contamination, each with a 1.9-m radius (0.3 to 1.7% contaminated area) and a hazard at 3 log(CFU/g). All plans collected 60 grabs of 25-g produce subsamples. (Right) Probability of acceptance for the same three sampling strategies applied to detect systematic contamination at −3 log (CFU/g) covering the whole field (100% prevalence). All plans collected 60 grabs of 2.5- to 20-g produce subsamples, leading to total sample masses from 150 to 1,200 g. Each scenario was iterated for 100 contamination patterns and 100 sampling patterns (*n* = 10,000 iterations). Boxplots show median lines, the IQR, upper whiskers (calculated as Q3 max value + 1.5× IQR), lower whiskers (calculated as Q1 min value − 1.5× IQR), and outliers as points.

Overall, the median acceptance probability decreased from ~100% to 63% as the prevalence increased from 0.3% to 1.7% when taking 60 grab samples of 25 g in different patterns (Fig. 4, left). Specifically, SRS and STRS exhibited similar trends, with the median acceptance probability decreasing from ~90% to ~63%. Both sampling strategies resulted in similarly small variability in acceptance probability, suggesting stable sampling performance. By contrast, while k-step SS showed decreasing median acceptance probability, it decreased from ~100% to ~75%. In addition to its higher acceptance probability, k-step SS exhibited a substantially larger variability, especially when the prevalence was above 0.8%, indicating unstable sampling performance. In conclusion, SRS or STRS performed better than k-step SS for detecting a point source contamination, due to less variability in performance.

### All sampling plans showed similar performance to detect widespread contamination scenarios, but the power was dependent on total sample mass.

By visual inspection, the three sampling strategies had similar performance (Fig. 4, right). The median acceptance probability decreased from ~85% to ~30% as the total sample mass increased. In addition, all sampling strategies had relatively small variability in acceptance probability. In conclusion, under systematic, widespread contamination, sampling performance was dependent on the total sample mass, regardless of sampling strategy.

Based on the first two simulations, it was evident that SRS and STRS had similar sampling performance, which was superior to that of k-step SS. Therefore, for simplicity, we evaluated only SRS in the subsequent power analysis simulations in fields with industrially relevant, rare contamination.

### Sampling and testing a 60-grab sample will not reliably identify a 1-acre field with a single point source of contamination.

**FIG 5:**
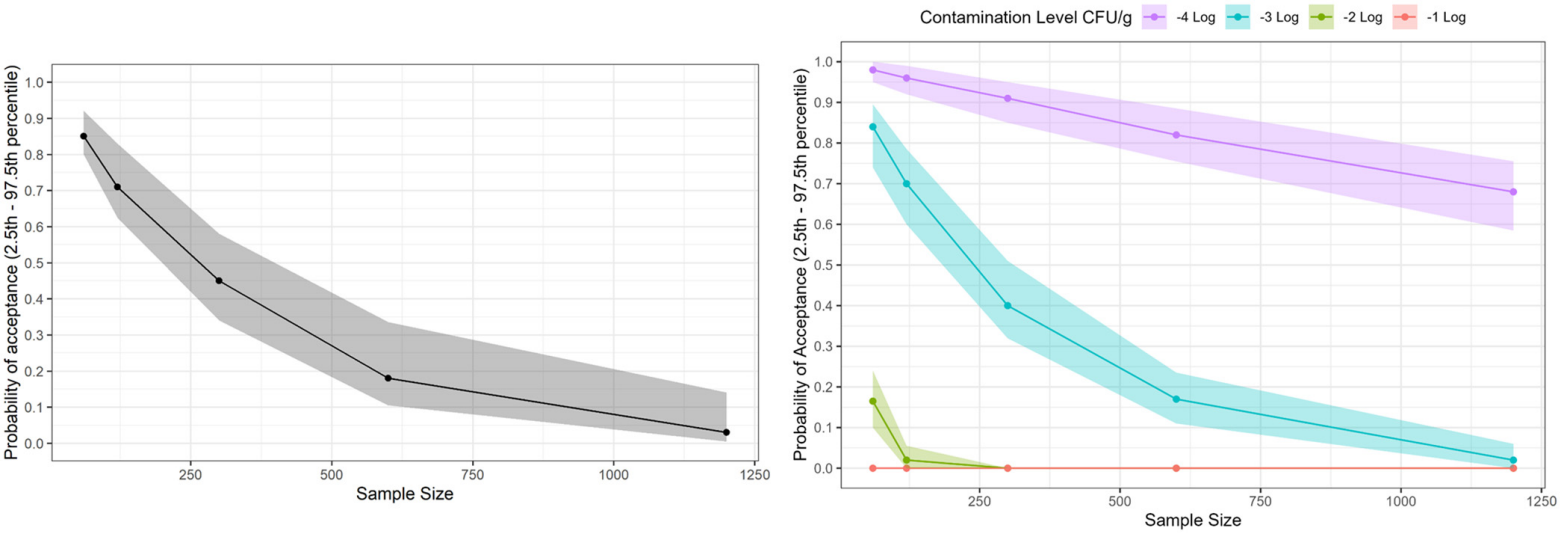
Analysis of the performance of simple random sampling plans with increasing sample sizes (and therefore masses) to detect representative hazards in 1-acre generic fields. (Left) Probability of acceptance for detecting a point source of contamination, such as a single fecal pellet (with 1.9-m radius spread [12], 0.3% of field), at 3 log(CFU/g), taking different numbers of produce samples from 60 to 1,200 samples of 3 g each. (Right) Probability of acceptance for detecting systematic contamination in a field with relatively low-level contamination [−4 log(CFU/g), or 1 CFU/10 kg of product] to high-level contamination [−1 log(CFU/g), or 1 CFU/10 g of product], taking 60 to 1,200 produce sample grabs of 3 g. Simple random sampling was applied in both simulation experiments. Probabilities of acceptance are presented as medians and 2.5th to 97.5th percentile intervals. Each scenario was iterated for 100 contamination patterns and 100 sampling patterns (*n* = 10,000 iterations).

Typical industry sampling with 60 produce sample grabs resulted in a median acceptance probability of ~85% (Fig. 5, left). As the sample size increased to 1,200 produce sample grabs, the median acceptance probability decreased from ~85% to ~5%, with similar variability. These results suggested that when the prevalence is low, the commonly used 60-produce sample grabs sampling plan may not be powerful enough to detect contamination. It would require a substantial number of additional samples to reject a contaminated field.

### Increasing the sample mass by collecting more grab samples might reliably detect high-level widespread contamination.

When the contamination level was relatively high [−1 log(CFU/g)], the median acceptance probability was 0% for all sample sizes, suggesting they all would detect contamination and reliably reject the field (Fig. 5, right). As the contamination level was reduced [from −1 to −4 log(CFU/g)], the probability of acceptance of the 60-grab, 3-g sampling plan increased from ~0% to ~100%, suggesting that lower contamination levels are not reliably detected using typical sampling strategies. Taking more grab samples increased the total sample mass collected, from 180 g (60 produce sample grabs of 3 g each) to 3,600 g (1,200 produce sample grabs of 3 g each), and decreased the probability of acceptance at all contamination levels. Figure 5 (right) shows that the larger total sample mass achieved by taking 1,200 samples in a field with −3 log(CFU/g) contamination had a probability of acceptance of 5%, while the same number of samples in a field with −4 log(CFU/g) had a probability of acceptance of 67%. These results suggest that when systematic contamination is relatively high, relatively low total sample mass will detect the contamination, but that much greater total sample mass is required to have even a modest probability to detect relatively low levels of systematic contamination.

## DISCUSSION

Here, we developed a preharvest sampling simulation for pathogen detection when hazards are present as point source, widespread systematic, or sporadic contamination in leafy greens fields. Overall, our findings show (i) this preharvest sampling simulation can predict experimentally observed sampling results, (ii) preharvest sampling plans with more sample points, collected with randomization, have greater probability to detect point source contamination, and (iii) preharvest sampling plans with larger total sample mass have greater power to detect widespread, low-level contamination regardless of the sampling pattern.

### This preharvest sampling simulation can reproduce previously published theoretical detection probabilities and predict experimentally observed sampling results.

The preharvest sampling simulation model successfully reproduced theoretical detection probabilities from a leafy green sampling study by Xu and Buchanan (19) by simulating equivalent conditions and generating probabilities of detection that matched published detection probabilities. The general conclusions of this validation exercise were consistent with those of the previous authors. Preharvest sampling with sample sizes of ≤30 showed low power to detect low-prevalence (0.3%) contamination in fields. Xu and Buchanan evaluated a Z-shaped, systematic sampling pattern, while we evaluated a serpentine pattern (k-step systematic sampling). In both cases, the systematic sampling performance was more variable than sampling with randomization. Combined with previous studies (11, 19, 26, 27, 28), our data show that computational models provide a powerful approach to assess food safety sampling strategies.

The preharvest simulation was also validated by field trials, which provided a simulated range of positive samples for each trial that included the number of positive samples obtained in each trial. This exercise showed it was important to account for reduction in pathogens from the time of contamination (here, inoculation) to sampling. Xu and Buchanan also observed changes in bacterial concentration following inoculation in their validation trials (19). Our study accounted for these changes by developing a separate Monte Carlo simulation of the bacterial concentration change from inoculation until the day of sampling, separately accounting for adhesion (estimated from data) and die-off over time (highly variable, based on literature). The large variability in predicted pathogen concentration when sampling 5 to 6 days postinoculation (compared to 1 day postinoculation) suggests that one way to improve the reliability of preharvest testing is to sample as soon as possible following any observed potential contamination event. Granted, only some contamination events in a well-functioning system would be observed, e.g., a rainfall event close to the harvest date, whereas others would be unobserved, e.g., unknown factors.

### Preharvest sampling plans with more sample points, collected with randomization, have greater power to detect point source contamination.

The purpose of developing the preharvest sampling simulation was to provide scientifically validated sampling guidance for application to commercial testing on leafy greens fields. Therefore, this study assessed sampling to detect realistically low-prevalence or low-level contamination in 1-acre fields. Our simulations suggest that sampling plans with more sample points, collected with some randomization (e.g., simple or stratified random sampling), are better for detecting point source contamination. Here, “better” means less variable performance, and under some conditions, increased power.

In terms of the sampling pattern performance, these results were consistent with the findings of Xu and Buchanan (19), that sampling plans with randomization had less variance than Z-pattern sampling in terms of the power to detect point source contamination in leafy greens fields. These results are also consistent with prior work from our group, in which we studied sampling to detect aflatoxin in corn (28). In that simulation, to evaluate the performance of randomized and systematic probe sampling in corn bins, systematic sampling had a much larger variability in performance and lower power, compared to randomized sampling when contamination was highly clustered (28), where clusters of aflatoxin contamination were analogous to point source contamination in a field. Further, taking 100 probe samples dramatically outperformed taking 5 or 10 probe samples in detecting the same highly clustered contamination.

One interesting, initially conflicting result came from work on theoretical statistics of food sampling applied to localized (point source) contamination in powdered infant formula (18, 29). That work showed that systematic sampling was at least as powerful as simple random sampling, and potentially up to 37% more powerful, to detect point sources of contamination in batches of powder, although for both sampling patterns they also found that increasing sampling numbers from 10 to 200 dramatically improved probability of detection. However, there are a few important differences between the two approaches. Their model of small point sources that contaminated all product in a short time range (e.g., 5 min) would be like contaminating an entire produce bed (whole row or column of a two-dimensional [2D] field). Our scenarios did not model this type of post-source contamination. Instead, we modeled radial spread from a point that only partially contaminated any given dimension. The powdered infant formula work used binomial and Poisson distributions to model systems and calculate detection probabilities. It did not predict variability in sampling plan performance, as does the Monte Carlo simulation used in this study and others (28). Our work suggests the main value of randomization is to reduce variability of performance. Finally, the authors of the powdered infant formula sampling study (29) did mention a caveat that systematic sampling could miss systematic contamination and that stratified random sampling would be better to detect such contamination. This suggests stratified random sampling may be an optimal sampling pattern across both random and systematic point source contamination. Considering the sampling performance results from studies across multiple commodities, sampling plans with larger numbers of samples collected with randomization, particularly stratified random sampling, outperform typical industry sampling plans in detecting point source contamination.

### Preharvest sampling plans with larger total sample mass have greater power to detect widespread, low-level contamination regardless of the sampling pattern.

This study also predicted that total sample mass collected determines the power of the sampling plan to detect widespread, low-level contamination, not the distribution of the sampling points, i.e., total mass matters, not the pattern. Intuitively, this makes sense. If contamination is widespread, then many samples should be taken from the contaminated zone (pattern won’t matter). But, if contamination is at a low level, those samples need to be large enough to actually capture a rare pathogen. These findings are consistent with the results presented by Hoelzer and Pouillot (30), who found that sampling plans with large sample numbers and large physical mass per sample (25 g), hence a larger total sample mass, are needed to detect foodborne pathogens at low prevalence (0.1%) and at −2 log(CFU/g) uniformly distributed in a batch of food. This also implies that some typical industry plans that comprise 60 produce sample grabs, ranging from 2.5 to 6.25 g of individual mass per grab, for a total of 150 to 375 g per composite, might fail to detect contamination; these plans contrast with the classic ICMSF Case 15 N60 sampling plan recommended for a broad range of higher-risk commodities; this plan requires 60 sample grab samples of 25 g each, for a total sample mass of 1,500 g, suitable for sampling a broader range of foods lots (8). It is important to note that in many of these analyses with a fixed subsample (i.e., produce sample grab) mass, more samples were directly correlated with greater total sample mass, providing dual benefits.

### Comparing this simulation to the ICMSF sampling plan tool.

The ICMSF sampling plan tool (31) is a respected resource for calculating power of sampling plans based on assumptions like prevalence, level, and standard deviation of contamination in lots and for taking a given number of samples of a given mass. The way our simulation models the contamination distribution differs from the ICMSF tool, while producing similar results under assumptions that create similar food systems.

Our model simulates a contamination event as having a mean and deviation and draws a value for contamination at each uncertainty iteration that is applied to all samples taken from the field. In the field test, positive is determined by the expected number of cells in each sample. If the expected number of cells is >1, the sample is positive. If the expectation is <1, the sample has a chance of being positive equal to the concentration, with status (positive or negative) drawn from a binomial distribution. Variability iterations determine the probability of testing positive. In contrast, the ICMSF tool treats the product lot as having variability in contamination defined by a mean and deviation, such that all individual samples drawn from a lot have a different level of contamination. The probability that a lot tests positive is determined by the probability that the required number of samples has the expected number of cells of >1.

Despite these differences, the ICMSF tool can be parameterized to represent a similar system as modeled in this study, and in that case will give similar results. The tool outputs 30% as the probability of acceptance when using the inputs for a sampling plan of 60 samples of 20 g each, (1,200 g total), with a mean of −3.00 log(CFU/g) and standard deviation of 0.05 log(CFU/g) (lowest deviation possible). This approximates our second simulation, which gave a median probability of acceptance of 28%. These values are for contamination events with low variability, which we used to illustrate a known food safety hazard.

One may also be interested in modeling conditions with increased variability. Simulation of contamination events with concentration distributions with the same mean [−3 log(CFU/g)] but larger variability [standard deviation of 1 log(CFU/g)] (data not shown), results in a probability of acceptance that ranges from 0% to 100%. In contrast, the ICMSF sampling tool outputs 0.14%. This illustrates the difference in the two approaches. Is the field contaminated by an uncertain event (our approach), or a certain event with variability (ICMSF approach)? There is clearly an opportunity for future work to incorporate both uncertainty in the contamination events and variability in the contamination profile. However, any model would then need (currently unavailable) data on the likelihood of various contamination events and the variable of contamination, in real production systems.

### Conclusion.

A preharvest sampling simulation was validated and then used to develop guidance for sampling plans for more powerful detection of foodborne pathogens. Taking a large number of samples, ideally with stratified randomization, improves detection of point sources of contamination. A larger total composite mass improves detection of systematic, large-area contamination events. Therefore, one can increase the power of preharvest sampling by taking more, smaller samples with randomization, up to the constraints of total produce sample grab number and total mass determined either by feasibility or by a predetermined food safety objective.

Further research could validate the conclusions from this study by sampling commercial leafy green fields with real-world contamination events, although it would be challenging to power and conduct such a study due to (fortunately) rare pathogen contamination. Also, technical approaches to collect more representative samples should be explored, since collecting hundreds of grab samples from a field is not likely feasible.

## MATERIALS AND METHODS

### Produce field simulation.

A simulation model for preharvest field sampling was adapted from the three dimensional corn bin model published by Cheng and Stasiewicz (28) to simulate the process of taking samples from a contaminated 2D field and testing samples for pathogens. For adaptation, this project recoded the portions of the model that operate in a 3D space to function in a 2D space, then changed input parameterization as needed. For example, in the 3D system, mycotoxin contamination clusters in a bin were centered around points in 3D space (*x*, *y*, *z*) with individual contaminated kernels placed randomly around the cluster. In the 2D system, point sources of contamination in a produce field are identified by the point source center (*x*, *y*) and a radius (*r*) of spread with all produce inside the radius affected. The core simulation consists of four major modules, where the hazard and sampling simulations were recoded, but the assay and decision-making simulations remain unchanged.

The first module is the field and hazard profile simulation module, where either point source or area-based contamination is simulated in a specific field. In this section, the end user can define the field dimensions, spatial pattern of contamination (i.e., as point source or covering the field area), and concentration of the hazard of concern (as a distribution).

**FIG 6:**
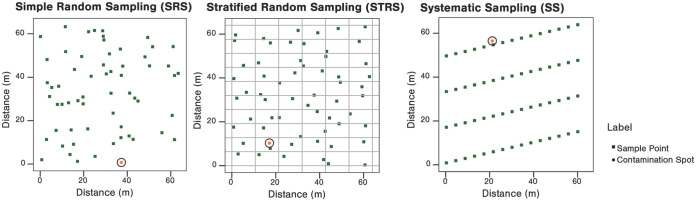
Simulated preharvest sampling example of 60 produce samples in a generic, 64- by 64-m (~1-acre) leafy green field with point source contamination at 0.3% prevalence. For simple random sampling (SRS), all simulated samples were randomly collected across the total simulated leafy green field. For stratified random sampling (STRS), the simulated leafy green field was divided into 60 strata (boxes). Each stratum was randomly sampled for one individual simulated sample. For systematic sampling (SS), simulated collected samples were spaced out evenly among the simulated leafy green field. A simulated sample was collected within the contaminated area using SS.

The second module is the sampling module. The end user can determine the sampling approach, i.e., determine the number of samples, their mass, and their locations in a contaminated field, using either simple random sampling (SRS), stratified random sampling (STRS), or k-step systematic sampling (SS) (Fig. 6). Strata for STRS can be defined by rows, columns, or their combination, where the combination creates “boxes” of sampling strata within a field. This implementation of SS spaces samples an even distance from each other (k-steps), by a single dimension (row or column), as if one were taking samples every given number of steps while walking up successive rows in a field, similar to an industry serpentine pattern. Once the hazard profile and the sampling locations are determined, the assay module will simulate the plating or enrichment process to quantify the results for each simulated sample.

After the sampling and assay modules, the decision-making module will decide whether to accept or reject the entire field. In this module, the end user may choose from the 15 different ICMSF attribute sampling plans (7), depending on the severity of the hazard. For instance, Case 15 of the ICMSF sampling plans rejects a field if any of the 60 produce sample grabs of 25 g are positive following enrichment, which means that there was ≥1 cell per 25 g. The user can also specify a custom plan with a customized number of produce sample grabs, masses, and rejection decision thresholds. The simulation model can be iterated to create a distribution for the probabilities that at least one sample will be collected from a contaminated section of the field regardless of assay results (probability of detection), that contamination will be detected if it is present (probability of detection), and that a field with contaminated produce will be accepted for harvest and sale (probability of acceptance). This definition of probability of detection is consistent with the terms used in the study selected for model validation (19). Since a field is rejected when the simulated assay result for the composite samples is above the rejection threshold (e.g., ≥1 CFU per total mass), fields with low levels of contamination could have samples taken within a contaminated zone (a positive probability of detection result), but those samples may not contain a bacterial cell and therefore would test negative. The field would therefore not be rejected (a positive probability of acceptance result). Detailed definitions for all the input parameters are listed in Table S1 in the supplemental material.

### Simulation validation against peer-reviewed literature.

Validating the simulation model with literature data involved three steps: (i) extracting the input values from the literature data and creating equivalent input parameters for our simulation model; (ii) iterating the simulation model to create a range of probability of detection outputs; (iii) comparing the probability of detection against the literature detection probability and determining whether the literature output fell within the relevant simulation output range. Specifically, our simulation model was considered validated when the detection probability from the literature data fell within the range of the 2.5th to 97.5th percentile of the simulated probability of detection; this assumes that the model is sufficiently iterated to generate all likely values of probability of detection.

The study reported by Xu and Buchanan (19) was used as literature validation source. This study simulated the sampling process in an 18- by 15-m field with 270 square subplots of 1 by 1 m. The study reported the detection probability under a combination of inputs, including 4 different numbers of samples (5, 10, 15, and 30) and 10 different numbers of contaminated subplots (from 1 to 10). Successful detection was defined as sampling at least one of the contaminated subplots in a field.

The first step to simulating that work was to represent the equivalent of their 1 by 1-m square contaminated subplots as circular point sources in our model. To do so, we set the point source spread radius to 0.56 m, so that each point source would have an area of 1 m^2^, matching that of Xu and Buchanan (19). We then mapped all relevant field and sampling parameters to the simulation model, to generate an equivalent 18 by 15-m field as well as sampling plans with 5 to 30 subsamples and 1 to 10 contaminated subplots. After parameter mapping, we iterated the model for 100 hazard contamination iterations and, for each of those, 100 sampling location iterations (*n* = 10,000 total iterations). These iterations were then used to generate the distribution used to calculate the probability of detection.

### Preharvest sampling plan simulation for hazard detection in generic 1-acre fields.

After validation, we used the simulation model to address industry-relevant sampling questions. Four simulations were conducted in 1-acre generic fields (i.e., a simulated general leafy green production field) using the input values specified in Table 2.

**TABLE 2:** Simulation model input variables for generic 1-acre field

Simulation no.	Contamination geometry	No. of contamination points	Hazard contamination distribution [log(CFU/g)]	No. of samples (*n*)	Sampling strategy^*a*^	Individual sample mass (g)	No. of iterations
1^*b*^	Point source (spread radius, 1.9 m)	1, 2, 3, 4, 5, 6	Normal (μ = 3, σ = 0.01)	30	SRS, STRS (strata = 5), SS	25	100 contamination events × 100 samplings/event
2^*c*^	Area	Not applicable	Normal μ = −3, σ = 0.01)	60	SRS, STRS (strata = 5), SS	2.5, 5, 10, 20	100 contamination events × 100 samplings/event
3	Point source (spread radius, 1.9 m)	1	Normal (μ = 3, σ = 0.01)	60, 120, 300, 600, 1,200	SRS	3	100 contamination events × 100 samplings/event
4	Area	Not applicable	Normal (μ = −4, −3, −2, −1; σ = 0.01)	60, 120, 300, 600, 1,200	SRS	3	100 contamination events × 100 samplings/event

a SRS, simple random sampling; STRS, stratified random sampling; SS, systematic sampling.

b Thirty samples of 25 g would be equivalent to ICMSF case 14.

c Sixty samples of 25 g would be equivalent to ICMSF case 15.

The first simulation focused on the relationship between sampling patterns and ability to detect point source contamination. In this case, we assumed point source contamination existed in a 1-acre field (64 by 64 m) and compared the sampling performance between SRS, STRS, and k-step SS under scenarios with 1 to 6 contamination points of 1.9-m radius each (12). These contamination points covered 0.3%, 0.6%, 0.8%, 1.1%, 1.4%, and 1.7% of the total area of the field, and essentially represented a prevalence of contamination.

The second simulation was designed to investigate the effect of individual sample mass and sampling strategy on sampling performance. For this simulation, we assumed a 1-acre field with widespread, systematic contamination (i.e., spread uniformly across the whole field area) and compared the probability of acceptance under the three sampling strategies taking 60 grab samples using 4 different subsample sample masses (2.5, 5, 10, and 20 g), leading to total samples masses of 150 to 1,200 g.

The third and fourth simulations were designed to evaluate the power of the optimal sampling patterns identified in simulations 1 and 2 to detect point source or systematic contamination at industry-relevant scales. These simulations explicitly tested the effect of increasing the number of grab samples beyond current industry practices and on the detection of the low levels of contamination that are expected in real-life produce fields. Based on the results of the first two simulations, we evaluated only the SRS sampling pattern, as it performed similarly to STRS and was superior to SS. Both the third and fourth simulations used 5 different sample numbers (60, 120, 300, 600, and 1,200 produce sample grabs of 3 g each). The third simulation evaluated a single point source of contamination (corresponding to 0.3% of the field area being contaminated) with a moderate contamination level [mean = 3 log(CFU/g), standard deviation (SD) of 0.01 log(CFU/g)]. The fourth simulation evaluated 4 different contamination levels (1 CFU per 10 g, 100 g, 1 kg, and 10 kg; i.e., −1, −2, −3, and −4 log(CFU/g), respectively, with SD of 0.01 log(CFU/g)] spread systematically in the field. We assumed low variability (i.e., SD of 0.01), because the purpose of these simulations was to illustrate the performance of sampling plans to detect a known food safety hazard, not to evaluate sampling plans under significant uncertainty (as would be typical in a quantitative microbiological risk assessment). For all the simulations, each sampling scenario was iterated for 100 contamination patterns and 100 sampling patterns (*n* = 10,000 iterations).

### Inoculated field trials setup.

**FIG 7:**
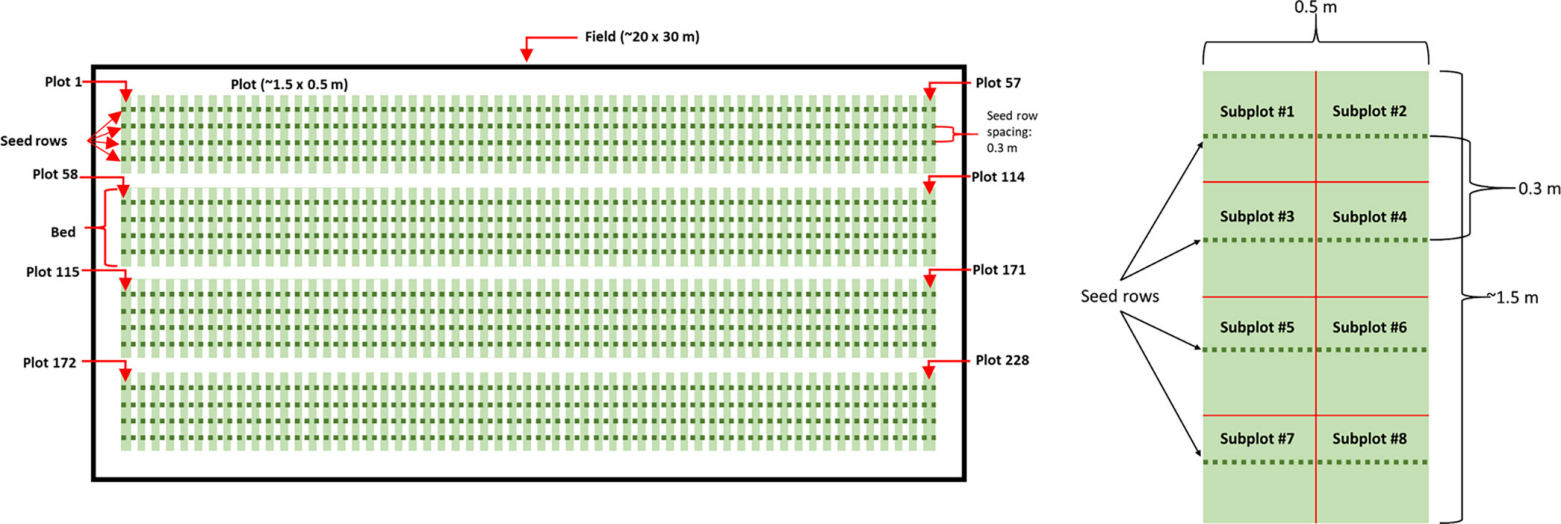
(Left) Overview of the field design for inoculated trials. Dimensions were approximately 20 by 30 m. Each field contained four beds planted with four rows of spinach. Each bed was divided into 1.5-m-wide, 0.5-m-long plots. Each bed was split into 57 identical plots, for a total of 228 plots across the four beds. (Right) Individual plot within bed. Each plot was subdivided into 8 subplots approximately 0.25 m long and 0.75 m wide.

Broadly, this aim involved creating inoculated field trials with controlled contamination events, followed by sampling those contaminated fields with predetermined sampling patterns, to generate experimental data that could be used to validate the simulation results. Briefly, four approximately 20-m by 30-m fields were planted with baby spinach (Seaside F1, treated seeds [Harris Seeds, Rochester, NY]) in Freeville, NY. Each field was made up of 4 beds, which were approximately 1.5 m wide and 30 m long; the beds were spaced approximately 1.5 m apart. Each bed had four rows of spinach planted at a seeding rate of approximately 2 cm. Each bed was split into 57 identical plots, such that each plot was 1.5 m wide and 0.5 m long; there were 228 total plots across the 4 beds (Fig. 7). Each plot was then split into 8 approximately equal-sized subplots. Irrigation, fertilization, and herbicide application were performed as needed.

Two of the four fields were used to investigate sampling plans following point source contamination, and the other two fields were used to investigate sampling plans following widespread systematic and sporadic contamination events. Six total trials were performed: two point source (PS1 and PS2), two systematic (SS1 and SS2), and two sporadic (SP1 and SP2) contamination trials. Systematic and sporadic contamination trials were performed in the same field but sampled at different days. Each field was independent of the others. For all contamination patterns, inoculation was performed 25 to 35 days following planting. Methods for inoculum preparation and controlled contamination were adapted from those reported in previous studies (2, 6, 12, 20). Briefly, each field was inoculated with a three-strain cocktail of rifampin-resistant generic E. coli (r*E.coli*; TVS 353, TVS 354, and TVS 355 (6, 20). Each of the strains was streaked onto two separate tryptic soy agar plates supplemented with 0.1 g/liter rifampin (TSA+R) to form a confluent lawn and incubated at 37°C for 18 to 24h. Following incubation, each plate was flooded with 10 mL of phosphate-buffered saline (PBS), the cells were resuspended using a sterile loop, and the resuspended cells were transferred into a sterile bottle containing 90 mL of PBS. The suspension in the bottle was vortexed. This process was performed for each plate, resulting in two separate bottles of 100 mL of suspension for each strain. From each bottle, 10 mL of each bacterial strain suspension was transferred to separate 15-mL conical tubes. The tubes were centrifuged at 4,000 rpm for 10 min, then the supernatant was pipetted off. The cells were then washed twice with PBS using the same centrifuge conditions as described above. The optical density (at 600 nm) of each suspension was measured to ensure the suspensions were at approximately 9 log(CFU/mL), based on a standard curve. The suspensions were then stored at 4°C overnight. The following morning, 4-mL aliquots of each strain’s suspension in each of the 6 bottles were combined, and serial dilutions were performed to reach the final concentration for inoculation.

For the simulated systematic and sporadic contamination events, the inoculum was diluted into 12 sanitized 2-liter bottles to reach a final concentration of 3 log(CFU/mL) PBS; 5 mL of each bottle was saved to confirm the inoculum concentration. Inoculation was performed using a CO_2_-powered backpack sprayer with pressure between 27 and 30 lb/in^2^ and two Turbo TeeJet (tip 8) nozzles spaced 38 in. apart. The inoculum was applied to the field at a rate of 66 mL per linear meter at a spray width of 3.76 m (±0.52 m), for an application of ~18 mL/m^2^, or ~4.2 log(CFU/m^2^). It was not feasible to directly calculate CFU per gram of plant because of all the uncertainty in the surface area of the plants, plant density, exposed soil, and other factors.

For the simulated point source contamination event, the inoculum was diluted into three 1-liter spray bottles with final concentrations of 0 log(CFU/mL), 3 log(CFU/mL), and 5 log(CFU/mL); a 10-mL aliquot from each bottle was used to confirm the inoculum concentration. To inoculate the field, a subplot within the field was randomly selected using a random number generator. A 2-m radius area was marked around the center of the selected subplot, and the area was split into three 0.67-m-radius rings. The inoculation of each ring consisted of 35 sprays (0.08 mL/spray) that covered 5 to 6 plants per spray, using a spray bottle. The inner, middle, and outer rings were inoculated with 2.8 mL of the 5-, 3-, and 0-log(CFU/mL) inoculum, respectively. This led to a sprayed inoculum per plant of ~3.2, ~1.3, and ~−1.1 log(CFU/plant) or, assuming ~5.4 g per plant, ~2.7, ~0.6, and ~−1.9 log(CFU/g) for the inner, middle, and outer rings. The inoculum was only sprayed on plants within the rings, not bare soil.

### Sample collection and sample testing.

The fields used for the simulated systematic contamination events were sampled 1 day following inoculation. These same fields were used to simulate sporadic contamination events by sampling 5 or 6 days following inoculation (trials 1 and 2, respectively), during which time some bacterial die-off should have occurred (4, 20, 32, 33). For the fields used for point source contamination, one sampling event was performed at 1 day following inoculation. At each respective sample collection, 228 samples were collected. Each sample consisted of all spinach plants in 1 of the 228 preselected subplots (1.5 by 0.5 m) per plot in the field; a random number generator was used to identify which subplot within each plot was collected. The spinach samples were collected by cutting each plant approximately 2 cm above the soil line using sanitized scissors. All plants within a single composite sample were transferred to a single Whirl-Pak bag (Nasco Sampling, Fort Atkinson, WI). The sample collectors’ gloves were changed, and the scissors were sanitized by wiping scissors blades with solution of 10% bleach followed by 70% ethanol between each sample. All samples were collected into a cooler with ice packs and transferred to the Cornell Food Safety Laboratory for storage at 4°C until processing.

All samples were processed within 24 h of collection. Each sample was weighed, and 225 mL of tryptic soy broth (TSB) was added. Each sample was hand massaged for 1 min, then incubated at 37°C for 18 to 24 h. After incubation, 50 μL of each enrichment was streaked for isolation onto E. coli CHROMagar supplemented with 0.1 g/liter rifampin plates (ECC+R; DRG International, Springfield, NJ). The plates were incubated at 37°C for 18 to 24h. ECC+R plates with blue colonies after incubation were recorded as being positive for rifampin-resistant E. coli and presumptive positive for the inoculated strains.

To determine if any of the r*E. coli* isolated from the spinach were naturally occurring rE. coli (i.e., not one of the inoculum strains), the PCR protocol described by Belias et al. (20) for differentiating the three inoculum strains (TVS 353, TVS 354, and TVS 355) was performed. For isolates that yielded a PCR banding pattern matching one of the inoculum strains, *clpX* PCR and subsequent sequencing was performed for further differentiation (20). Those isolates that did not match the TVS PCR banding pattern and did not have the same *clpX* allelic type were considered naturally occurring rE. coli and excluded from analyses. For the two simulated point source and sporadic contamination events, up to 2 isolates per positive sample were tested. As the two simulated systematic contamination events yielded a large number of positive samples, 2 isolates from 13 randomly selected positive samples were tested. This sample size was statistically sufficient to confirm <20% of the positive samples were positive due to naturally occurring rifampin-resistant E. coli at a 0.05 significance level (95% confidence and 80% reliability).

### Prediction of inoculum concentration on spinach at day of sampling.

In the process of simulating the inoculated field trials, we had to account for change in inoculum between spray inoculation, adhesion, and potential die-off in the 1 to 6 days between inoculation and sampling, consistent with observations that E. coli is known to die off in leafy greens over time (4, 20, 32, 33). Briefly, the structure of the model consisted of three main components: (i) inoculation; (ii) adhesion, and (iii) die-off. Each component was modeled to represent each stage of the field trial, taking the systematic contamination event 1 as a reference. For component (i), the sprayed inoculum concentration was assumed to be normally distributed around the inoculum concentration in the spraying bottles used at each trial, expressed in log CFU per milliliter. For component (ii), we modeled the fraction of inoculum that actually adhered to spinach as a reduction parameter fit to match the number of positive samples recovered in the systematic contamination trial (details are provided below). For component (iii), we used a model that represented a biphasic change in bacterial concentration over time after inoculation, based on data for New York State collected by Belias et al. (20). There was a rapid die-off (segment 1) and a slower rate (segment 2), separated by an inflection point (break point). Parameters were extracted from Belias et al. (20), filtered for region (New York) and commodity (spinach), log transformed, and subjected to distribution fit. The fitted die-off model was then used to predict total die-off over time between inoculation and sampling. Bacterial concentration at sampling was predicted by adding the result of each individual component, as follows: bacterial concentration at day of sampling = sprayed inoculum + adhesion + die-off. The three components of the die-off model, extracted data, and user inputs were entered into Microsoft Excel (version 2202) using @Risk 8.0 software (Palisade Corporation) (Table 3) and iterated 10,000 times for each inoculated field trial.

**TABLE 3:** Overall die-off model input parameters for prediction of inoculum concentration on day of sampling

Component	Cell	Variable	Input distribution formula						Units	Source
			PS1	PS2	SS1	SS2	SP1	SP2		
Inoculation	A1	Sprayed concn	=@RiskNormal (5.12, 0.1)	=@RiskNormal (5.15, 0.1)	=@RiskNormal (2.91, 0.34)	=@RiskNormal (3.13, 0.05)	=@RiskNormal (2.91, 0.34)	=@RiskNormal (3.13, 0.05)	Log(CFU/mL)	This study
Adhesion	A2	Log reduction after spraying and adhesion	=@RiskNormal (−2.975, 0.1)						Log(CFU/g)	Calculated
	A3	Concn adhered	= A1 + A2						Log(CFU/g)	Calculated
Die-Off	A4	Transformed breakpoint	=RiskExtValue (−0.45175, 0.26206, RiskCorrmat [breakpoint_segments])						Log days	20
	A5	Breakpoint	= 10^A4						Days	Calculated
	A6	Transformed segment 1 die-off rate	=RiskTriang(−0.9704, 1.0179, 1.0179, RiskCorrmat [breakpoint_segments])						Log(CFU/day)	20
	A7	Segment 1 die-off rate	= −1 × 10^A6						Log(CFU/day)	Calculated
	A8	Transformed segment 2 die-off rate	=RiskNormal (−0.17916, 0.20036, RiskCorrmat [breakpoint segments])						Log(CFU/g)	20
	A9	Segment 2 die-off rate	= (10^A8) − 1						Log(CFU/day)	Calculated
	A10	Days after inoculation	1	1	1	1	6	5	Days	User input
	A11	Log reduction at day of sampling	= IF(A11>A5, [(A7 × A5) + A10(A11 − A5)], [A11 × A7])						Log CFU	Calculated
Output	A12	Concn at day of Sampling	= A1 + A2 + A12						Log(CFU/g)	

### Calculation of the adhesion parameter.

In the process of simulating the inoculated field trials, we needed to account for change in concentration between spraying inoculum and what adhered onto field spinach. This was because initial simulation of the systematic contamination trials using the sprayed inoculum [~3 log(CFU/mL)] and adjusting only for 1 day of die-off predicted that all 228 samples would be positive in all iterations (data not shown), which was not consistent with the 139 positive samples recovered from those trials. To obtain this parameter, we calculated the adhered inoculum concentration needed to recover 139 positive samples at time of sampling, as observed in the systematic contamination trials. This concentration was back-calculated from the following equation:

(1) $$E\left(n_{+}\right)=n_{\mathrm{sp}}\times {\text{CFU}}_{i}=n_{\mathrm{sp}}\times m_{\mathrm{sp}}\times C_{i}$$

where *E*(*n*_+_) is the expected number of positive samples, *n_sp_* is the total number of samples, CFU*_i_* is CFU in sample *i*, *m_sp_* is sample mass (in grams), and *C_i_* is the inoculum adhered to sample *i* (in CFU per gram).

In this case, we assume *E*(*n*_+_) follows a binomial distribution with *n* = *n_sp_* and *p* = CFU*_i_*. As contamination is extremely rare, we assume CFU*_i_* may fall within the range between 0 and 1, which can be interpreted as observing one positive sample in every 1/CFU*_i_* samples under enrichment. For example, 0.001 CFU can be interpreted as detecting 1 CFU present in 1 of every 1,000 samples, leading to 1 positive sample in 1,000 samples. We recognize this approach treats a cluster of contamination in a sample the same as a single cell. With the goal of back-calculating *C_i_*, we transformed equation 1 and assigned experimentally acquired values to all other parameters:

(2) Ci=E(n+)nsp×msp=139228×18.2 g≈−1.47 log ⁡CFU/g

With the sprayed inoculum concentration provided and the adhered inoculum concentration calculated, we estimated the inoculum concentration reduction during the adhesion process to be −2.98 log(CFU/g).

### Simulating inoculated field trial results.

Validating the simulation model with the experimental data involved four steps: (i) We created a distribution for hazard at time of sampling. This involved selecting the best fit distributions for hazard concentration at sampling day, as defined by lowest Akaike information criterion. (ii) Then, to reproduce that distribution in R (Table 1) using the package “ordinal” (34); we extracted the field and sampling parameters from the field trials and created equivalent inputs for the simulation model. (iii) We iterated the simulation model to create a range of probability of detection outputs and (iv) compared the simulation output with the field trials output and determined whether the field trial results fell within the simulation output range. Specifically, our simulation model was considered validated when the total number of positive samples from each trial fell within the 2.5th to 97.5th percentile range of the positive samples detected on each simulation.

We validated the simulation by producing results consistent with inoculated field trials. The inoculated field trials had three experiments: (i) a point source contamination event in two fields with sampling 1 day following inoculation; (ii) a systematic, high-level contamination event in two fields with sampling 1 day following inoculation; and (iii) a sporadic contamination event where the two fields sampled for systematic contamination were resampled at 5 or 6 days after inoculation, such that anticipated bacterial die-off created relatively low levels of contamination. Each simulated inoculated field trial was iterated for 100 hazard contamination iterations and, for each of those, 100 sampling location iterations were run (*n* = 10,000 total iterations).

### Open access tool developed for stakeholders.

The preharvest simulation is available as a web app for open access to industry, academic, or regulatory stakeholders. In this app, a user can analyze a scenario of their choosing, by specifying the field size, contamination pattern and level, and their specific sampling plan. The app can be found online at https://go.illinois.edu/foodsafetysampling2.
