# Supplementary material for: A Validated Preharvest Sampling Simulation Shows that Sampling Plans with a Larger Number of Randomly Located Samples Perform Better than Typical Sampling Plans in Detecting Representative Point-Source and Widespread Hazards in Leafy Green Fields
Source: Appl Environ Microbiol. 2022 Nov 15;88(23):e01015-22. doi: 10.1128/aem.01015-22 (PMC9746328; doi:10.1128/aem.01015-22)
Supplement: Supplemental file 1 — Supplemental material. Download aem.01015-22-s0001.pdf, PDF file, 0.2 MB [file aem.01015-22-s0001.pdf]

Supplemental Material

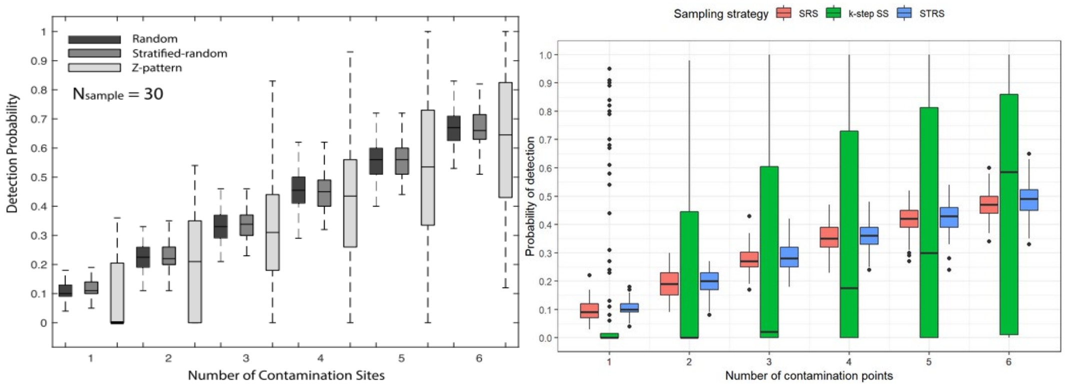

Figure S1. (Left) Detection probability versus number of contamination sites (1-6) with three sampling strategies (SRS, STRS, Z-pattern). Sample size is fixed at 30. Figure reproduced with permission from the publisher from (1). **(Right)** Probabilities of detection with a series of contamination points (1-6), a fixed sample size (30), and three sampling strategies (SRS, STRS, k-step SS). Sample size is fixed at 30.

Table S1. Simulation model input variable description.

Simulation Module	Variable	Description	Value range / Distribution	Default value	Unit	Source
Field and Hazard profile Module	x_lim, y_lim	Dimensions of the field (length, width)	> 0	1, 1	m	User input
	$geom$	Contamination geometry	point, area	point		User input
	n_contam	Number of contamination points (only applicable when $geom$ = 'point')	positive integer	3		User input
	$cont\_level$	Contamination level	log normal distribution (μ, σ)	μ = 3, σ = 1	Log (CFU/g)	Assumed
	bg_level	Background level	> 0	0.00001	CFU/g	Assumed
	$spread\_radius$	Spreading radius	> 0	1	m	Assumed
	LOC	Limit of contamination contribution (modifying decay function shape)	0 ~ 1	0.001		Assumed
	fun	Decay function of contamination level	exp, norm, unif	exp		User input
Sampling Module	$method\_sp$	Sampling strategy	SRS, STRS, SS	SRS		User input
	n_sp	Number of samples	5, 10, 15, 20, 30, 60, or other positive integers	60		(2)
	n_strata	Number of strata (for STRS)	positive integer	NA		User input
	by	Sampling direction (for STRS and SS)	row, column, 2d	NA		User input
	m_sp	Individual sample mass	> 0	25	g	(2)
Assay Module	$method\_det$	Detection method	plating, enrichment	enrichment	CFU/g or CFU	User input
Decision-Making Module	$case$	Attribute sampling plans	1 ~ 15	15		(2)
	m	Microbial count threshold (low)	≥ 0	0	CFU/g	(2)
	M	Microbial count threshold (high)	≥ 0	0	CFU/g	(2)

Table S2. Spearman correlation matrix for Die-Off rates and breakpoint in simulation model

@Risk Correlations	Segment 1	Segment 2	Break Point
Segment 1	1		
Segment 2	-0.19	1	
Break Point	-0.431	0.593	1

Table S3. Die-Off simulation model outputs and fitted distribution formula

Field Trial	Output Distribution Formula	Minimum	Maximum	2.5%	Median	97.5%	Mean	SD
PS1	@RiskExtvalueMin (0.98718, 0.9307)	-7.947	2.512	-3.374	0.747	1.987	0.401	1.409
PS2	@RiskExtvalueMin (1.0153, 0.92847)	-7.894	2.680	-2.266	0.781	1.907	0.429	1.409
SS1	@RiskExtvalueMin (-1.19, 1.0076)	-9.961	0.902	-5.825	-1.484	0.0117	-1.806	1.451
SS2	@RiskExtvalueMin (-0.99386, 0.92748)	-9.848	0.563	-5.374	-1.240	-0.0047	-1.580	1.409
SP1	@RiskNormal (-3.303, 2.093)	-9.987	8.012	-7.220	-3.434	1.232	-3.303	2.093
SP2	@RiskNormal (-2.8047, 1.8444)	-10.0	5.605	-6.607	-2.820	0.861	-2.805	1.844

References

1. Xu A, Buchanan, RL. 2019. Evaluation of sampling methods for the detection of pathogenic bacteria on pre-harvest leafy greens. Food Microbiol 77:137-145 doi:https://doi.org/10.1016/j.fm.2018.09.007.
2. International Commission on Microbiological Specifications for Foods (ICMSF). 2018. Sampling Plans, p 145-163 doi:https://doi.org/10.1007/978-3-319-68460-4_7. Springer International Publishing.
